# Energy harvesting from sonic noises by phononic crystal fibers

**DOI:** 10.1038/s41598-022-14134-9

**Published:** 2022-06-22

**Authors:** Farzaneh Motaei, Ali Bahrami

**Affiliations:** Optoelectronics and Nanophotonics Research Lab. (ONRL), Faculty of Electrical Engineering, Sahand University of Technology, Tabriz, Iran

**Keywords:** Structure of solids and liquids, Devices for energy harvesting

## Abstract

In this investigation, a phononic crystal-based fiber is proposed for energy harvesting application in metalworking factories. Phononic crystal plays the role of cladding in elastic fiber structure. Each of single-core fibers includes a tungsten hollow cylinder in central region which its internal radius is different in three single-core fibers. Incident waves with central frequency from 25 to 40 kHz of 1/3 octave band are confined in the core region of proposed elastic fibers and transmitted to desired distance. High confinement and transmission ability without significant longitudinal loss make this structure distinct from the other phononic crystals-based energy harvesters. By utilizing of a piezoelectric film at the end of fiber cores, mechanical energy is converted to electrical energy. As proposed elastic fibers confine the applied waves with high quality, the obtained output power is enhanced up to 800 times in comparison with the bare case. Maximum value of extinction ratio between all single core fibers is equal to − 23 dB. Also, longitudinal loss is almost equal to 0.9 dB/km.

## Introduction

Elastic fibers based on phononic crystals can be a novel proposal to be considered as the counterpart of photonic crystal fibers which may be used in many applications in acoustic/elastic field. Optical fibers as the important discovery in the communication field, have been innovated in the early twentieth century^1^. Traditional optical fibers or total internal reflection (TIR)-based fibers were transmitting the optical information with a very high speed and insignificant losses. Then, photonic crystal fibers (PCFs) were introduced in 1996 by Knight et al*.* for the first time^2^ which are classified into photonic band gap guiding and index guiding fibers^1^. Since photonic crystal fibers played an important role in the optical field, phononic crystal fibers have been introduced and investigated for the first time in our previous work^3^. In the previous study, an elastic fiber with a central core and a phononic crystal cladding in the surroundings has been investigated. Phononic crystals are a periodic configuration which have been utilized in acoustic field for controlling the acoustic/elastic wave propagation. Existence of scatterers in a background with diverse physical properties makes frequency band gaps in the phononic crystal dispersion curve^4^. Some of the phononic crystal applications are in acoustic waveguides^5^, material detecting sensors^6,7^, mechanical wave switches^8,9^, frequency filters^10,11^, energy harvesters^12–17^, demultiplexers^10,18^, and elastic fiber as a new device^3^.

Actually, phononic crystal bandgap in the elastic fiber confines the applied acoustic waves in the core region. Hence, this structure can be operated as a phononic band gap guiding fibers. Phononic crystal fibers with the initial idea of confining and guiding the elastic waves can be used in many acoustic applications in near future. One of the prominent application of the elastic fiber is electrical energy harvesting discussed in this paper. Elastic wave focusing in a small area (core) can be used for piezoelectric energy harvesting. Actually, resonance phenomenon makes elastic wave confinement in the core region of fiber. Besides, mechanical waves with high amplitude are used for electrical energy harvesting application. Energy harvesting is a technique for converting the ambient energy into useful energy. Electrical energy harvesting from mechanical energy such as vibrations and waves has been named as piezoelectric energy harvesting (PEH)^19^. There are many mechanical wave sources in the nature including ocean waves, sound, vibration, ultrasonic waves, human industrial activities, etc. Converting the mechanical energy into the electrical energy is done by piezoelectric materials such as polyvinylidene fluoride (PVDF) and lead zirconate titanate (PZT). A comprehensive review of piezoelectric energy harvesting devices have been done by Yang et al.^20^. In the last decade, utilizing of the phononic crystals for energy harvesting has attracted the attentions of researchers. From energy carrier point of view, phononic crystal energy harvesters can be divided into vibration, acoustic and elastic energy harvesters. The frequency of vibration carriers is less than that in acoustic/elastic waves^12–15^. Besides, according to the structure designing and working mechanism, phononic crystal energy harvesters based on locally resonant and defect states could be introduced. By removing one or more scatterers from perfect phononic crystal, defect state-based phononic crystal harvesters are formed. In locally resonant-based structures, ambient waves are focused in a special point^12,13^.

In 2013, a vibration energy harvester has been designed and fabricated based on two dimensional phononic crystal with point defect. Vibration waves with resonance frequency of point defect had been confined in the cavity. A piezoelectric film had been placed in the cavity to electrical power generation^12^. In 2015, vibration wave energy harvesting using a locally resonant phononic crystal plate with spiral beams was designed. Applied waves with low frequencies (0–500 Hz) have been confined in the central region of spiral beams. Output voltage in locally resonant region is 3 times of the other regions^13^. Tol et al. designed a gradient-index phononic crystal lens structure for energy harvesting performance. Piezoelectric device had been located in the focus point of the phononic crystal lens and hence the energy harvesting was realized^16^. Also, a two dimensional octagonal phononic crystal has been presented which confinement and localization of elastic waves were done in the point defect. By utilizing this phononic structure, energy harvesting power enhancement is achieved up to 22.8 times in comparison with metamaterial absence case^17^. Hyun et al.^15^, have designed and fabricated an omnidirectional gradient-index phononic crystal for acoustic wave focusing and energy harvesting. Ambient waves are confined in the central location of the structure and make the output power up to 4.5 times in comparison with the bare case^15^.

**Figure 1:**
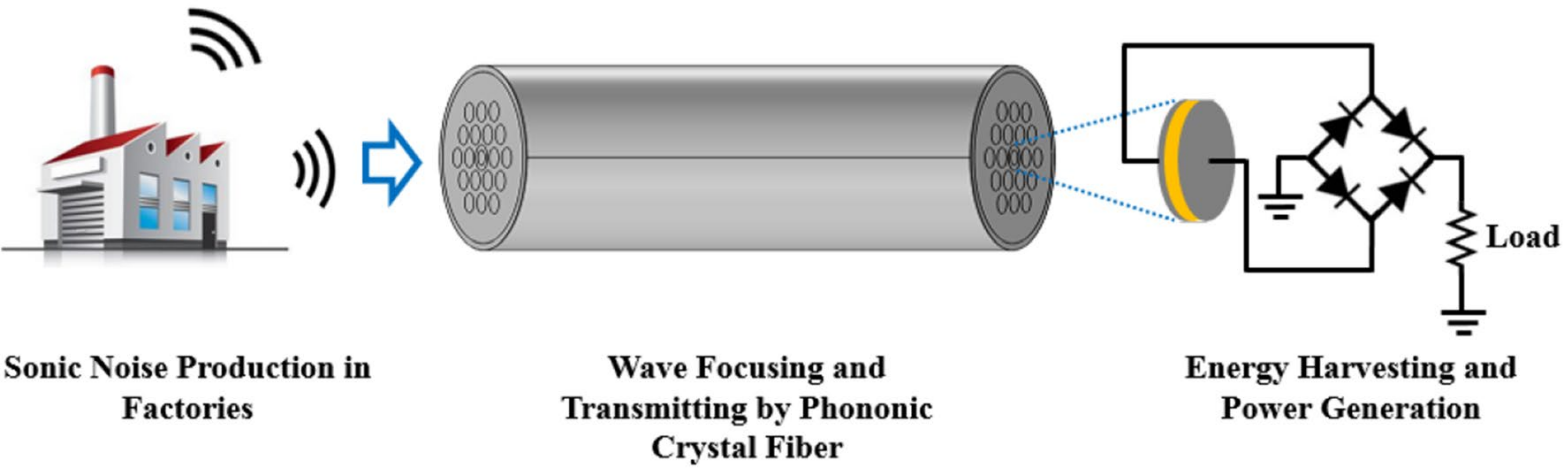
Power generation process by phononic crystal fibers.

In this paper, we have tried to propose an energy harvesting structure for confining the factory noises based on phononic crystal fibers. Metalworking factories which are related to cutting or sharpening of metals, produce many sonic noises with high frequencies. Usually, sound waves with frequencies above 20 kHz are defined as ultrasonic waves. The assessment of ultrasonic noise exposure is based on sound pressure levels in the 1/3 octave band (the central frequencies are in the range from 10 to 40 kHz). Also, in metalworking factories, the produced noise frequency range corresponds to the 1/3 octave band^21^. Thus, metalworking environment is a suitable source for production of ultrasonic waves. From this ultrasonic noise, energy harvesting can be realized. Through the phononic crystal fiber, ambient mechanical energy can be confined and transmitted to an electrical generation and storage station. Then, by a piezoelectric material placed at the end of fiber core, ac voltage is generated. Eventually, ac voltage is converted to dc voltage by rectifiers and capacitors and generated power can be saved. This process has been depicted in Fig. 1.

Some fundamental differences between phononic crystal fibers compared to the other energy harvesters based on phononic crystals are more confinement capability and ability of fiber to transmit the confined wave to the electrical storage station. On the other hand, the piezoelectric film and its electrodes are located outside the fiber structure and fabrication process becomes easier.

In this study, we have designed a phononic crystal fiber with different core sizes for central frequencies of 25 kHz, 31.5 kHz, and 40 kHz of 1/3 octave band because the maximum value of ambient sonic waves occurs in these frequencies^21^. At the end of elastic fiber, a PVDF piezoelectric film has been considered. The internal region of core is poly methyl methacrylate (PMMA) so PVDF has been selected to minimize the acoustic impedance difference between two materials.

Next items in following have been organized as the design procedure of phononic crystal fiber in “Design procedure of phononic crystal fiber” section, simulation results in “Simulation results” section, discussions about results in “Discussions” section, and conclusion of paper in “Conclusions” section.

## Design procedure of phononic crystal fiber

**Figure 2:**
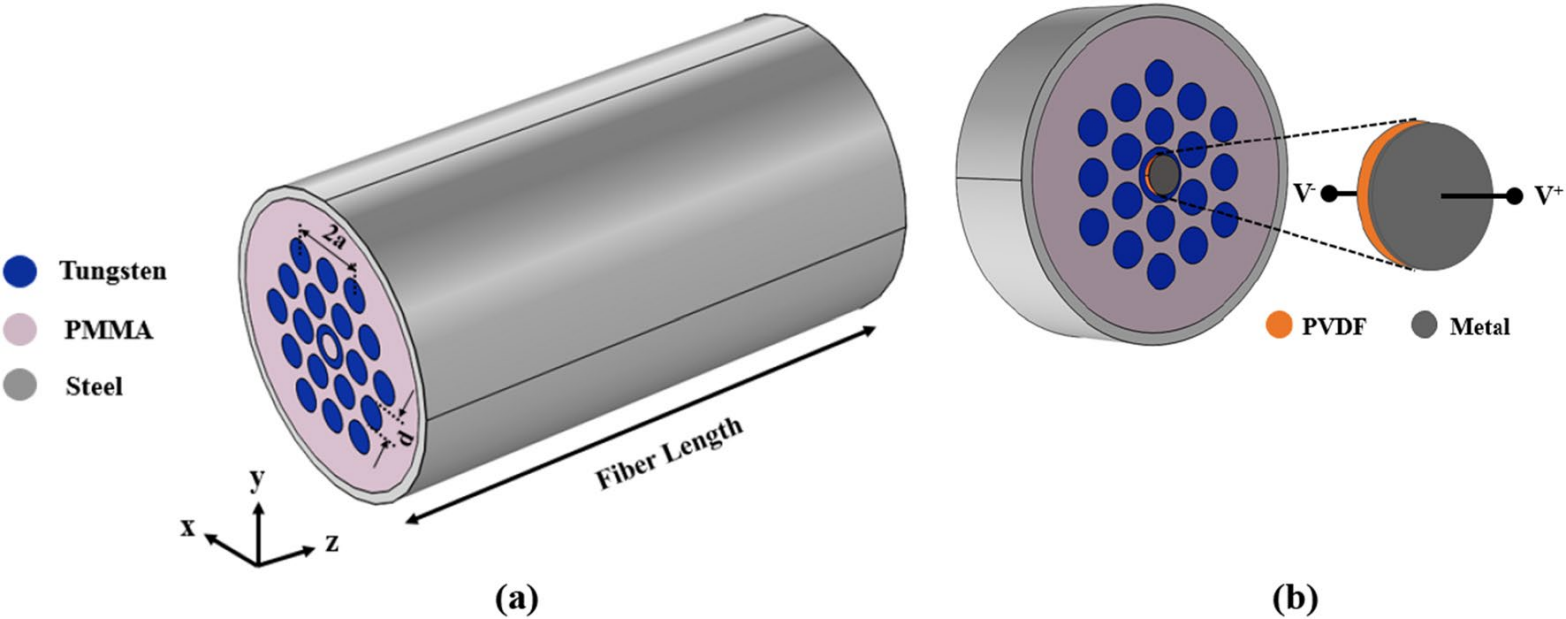
(**a**) Total schematics of the proposed phononic crystal fiber, and (**b**) piezoelectric location at the end of fiber core.

Phononic crystal in elastic fiber structure plays the cladding role and confines the incident waves in the core region. Hence, the frequencies of applied wave have to be placed in the band gap of surrounding phononic structure. The proposed phononic crystal fiber has a PMMA background which tungsten rods running along fiber length with hexagonal lattice arrangement. Also, a tungsten hollow cylinder has been embedded as core which has been depicted in Fig. 2.

Table 1 includes the elastic properties of constituent materials.

**Table 1:** Elastic properties of the used materials in elastic fiber/

Material	Mass density (kg/m^3^)	Young’s modulus (GPa)	Poisson ratio
PMMA	1190	3	0.35
Tungsten	19,350	411	0.28
Steel	7850	209	0.28

**Figure 3:**
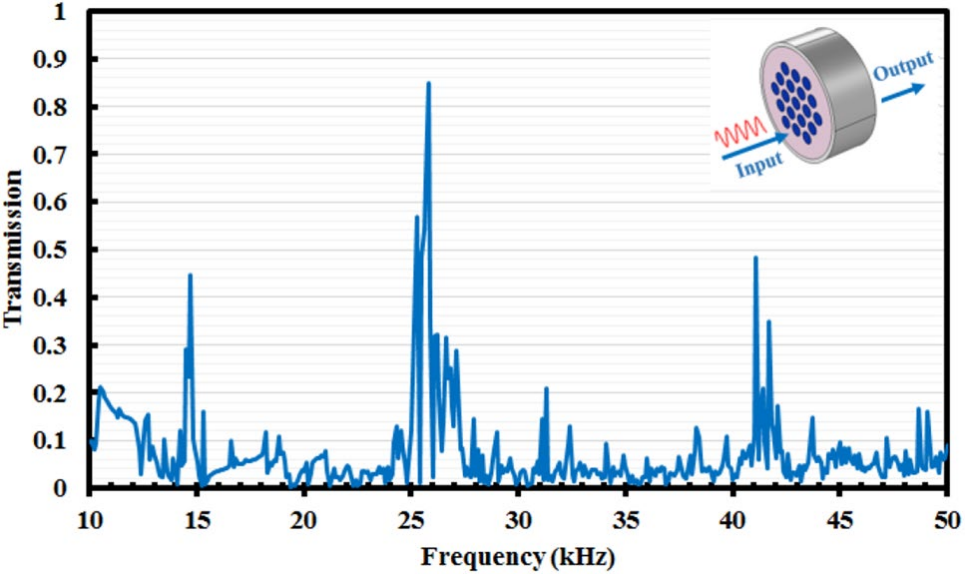
Transmission spectrum of proposed elastic fiber without core.

According to Fig. 2, diameter of scatterers is *d* and equals to 29.5 mm and lattice constant is *a* and equals to 39 mm. Incident waves are confined in the internal region of the tungsten hollow cylinder. The steel layer has been considered as cover. Transmission spectrum of proposed phononic crystal fiber without core has been achieved by a finite element method (FEM) software in the fiber length of 90 mm and its result has been shown in Fig. 3.

According to transmission spectrum, prohibited frequency bands are clear. Frequency of incident waves must to be placed in these prohibited regions. Proposed phononic crystal is solid–solid type so elastic wave propagation are described by following fundamental relations^4^:

1 $$T_{ij} ({\mathbf{r}},t) = c_{ijkl} ({\mathbf{r}})u_{k,l} ({\mathbf{r}},t)$$

2 $$T_{ij,j} ({\mathbf{r}},t) = \rho ({\mathbf{r}})\frac{{\partial^{2} u_{i} ({\mathbf{r}},t)}}{{\partial t^{2} }}$$

where *T*_*ij*_ is stress tensor and *u*_*i*_ is considered for displacement components in space. Also, mass density is *ρ* and elastic constants is *c*_*ijkl*_. Indices *i*, *j*, *k*, and *l* embrace values from 1 to 3 for three space components. A comma before an index carry the derivation concept (e.g., $u_{k,l} = \frac{{\partial u_{k} }}{{\partial x_{l} }}$), and summation over repeated indices is implied (e.g., $T_{ij,j} = \sum\nolimits_{j = 1}^{3} {\frac{{\partial T_{ij} }}{{\partial x_{j} }}}$). Combination of relations 1 and 2 results the below equation^4^:

3 $$(c_{ijkl} ({\mathbf{r}})u_{k,l} ({\mathbf{r}},t))_{,j} = \rho ({\mathbf{r}})\frac{{\partial^{2} u_{i} ({\mathbf{r}},t)}}{{\partial t^{2} }}$$

Equation (3) is utilized as without stress relation for modeling the solid–solid compositions.

## Simulation results

**Figure 4:**
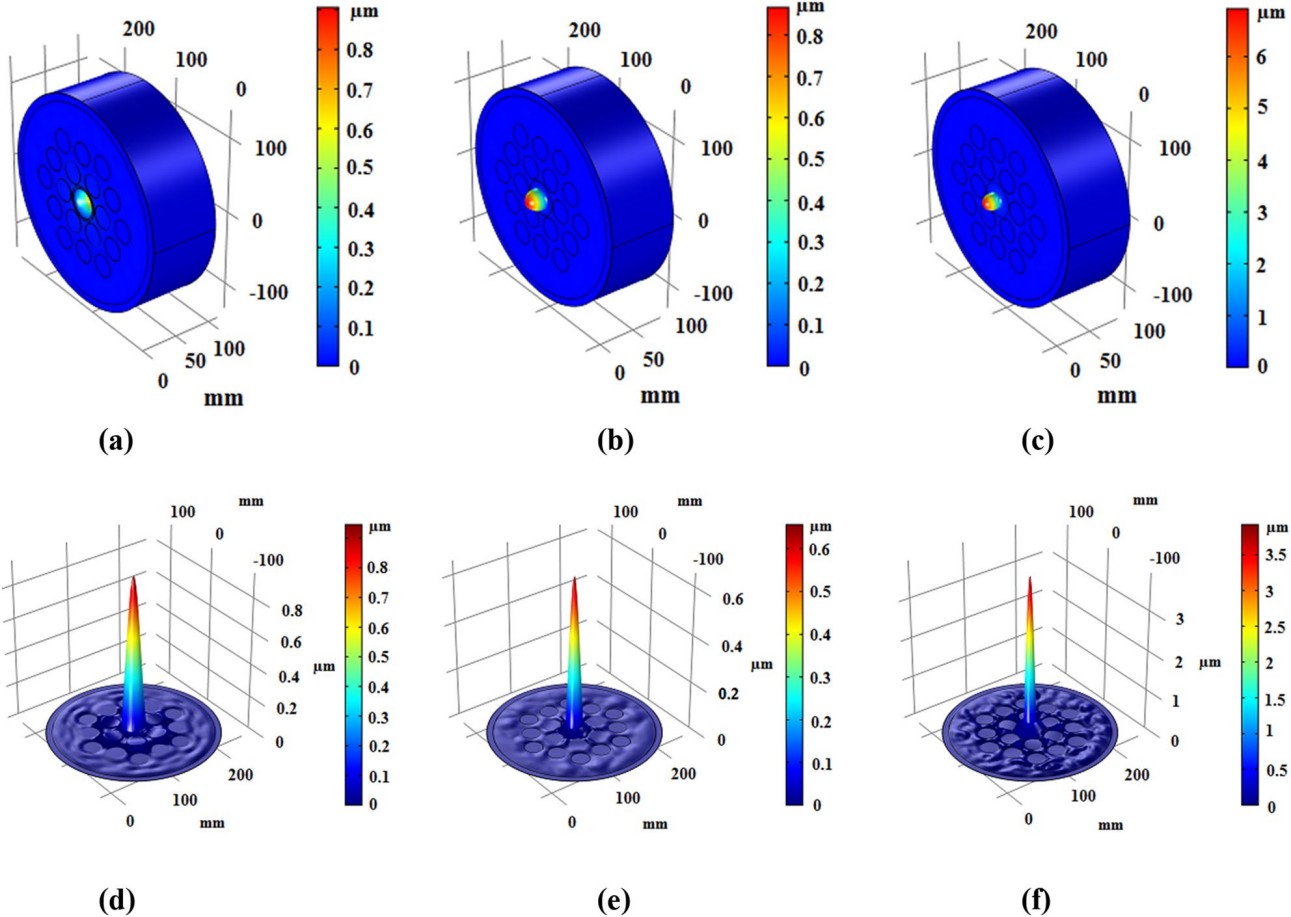
Displacement field of elastic wave in the phononic crystal fiber with internal radius of (**a**) r_1_, (**b**) r_2_, and (**c**) r_3_ and confinement of incident wave in cross-section of fiber and in length of 90 mm (end of fiber) for (**d**) r_1_, (**e**) r_2_, and (**f**) r_3_.

A new phononic crystal-based fiber for energy harvesting application was proposed in the previous section. In this section, simulation results achieved by a finite element method (FEM) software are presented. Regarding to central frequencies of 1/3 octave band (25 kHz, 31.5 kHz, and 40 kHz), wave intensity is almost equal to 100 Pa in metalworking factories^21^. In order to obtain high confinement for applied acoustic waves with frequency of 25 kHz, 31.5 kHz, and 40 kHz, we have optimized the internal radius of cores (*r*_*i*_). The designed internal radii are as *r*_1_ = 19.39 mm corresponding to 25 kHz, *r*_2_ = 13.68 mm corresponding to 31.5 kHz, and *r*_3_ = 9.78 mm corresponding to 40 kHz. In other words, these frequencies are resonance frequencies of different cores. In Fig. 4, propagation and confinement of incident wave with mentioned frequencies are depicted.

It can be seen from Fig. 4a which the incident wave with frequency of 25 kHz has been propagated with high confinement in the core. Also, applied wave with frequency of 31.5 kHz can be focused as shown in Fig. 4b. The proposed structure in Fig. 4c can confine the incident wave with frequency of 40 kHz, properly. Since the piezoelectric energy harvester has placed at the end of core, the displacement value at the end of fiber is important for this study. Displacement field and elastic wave confinement have been shown in Fig. 4d–f for frequency of 25 kHz, 31.5 kHz, and 40 kHz, respectively. In order to achieve the energy harvesting performance, a PVDF film with thickness of 250 µm has been considered at the end of fiber cores depicted in Fig. 2b. Incident waves with mentioned frequencies are transmitted and confined through the phononic crystal fibers so a piezoelectric energy harvester at the end of cores can convert the mechanical energy to electrical energy by direct effect of piezoelectric materials. The fundamental coupled equations to describe piezoelectric effect are as following:

4 $${\mathbf{T}} = s^{E} \cdot {\mathbf{S}} - d^{t} \cdot {\mathbf{E}}$$

5 $${\mathbf{D}} = d \cdot {\mathbf{S}} + \varepsilon^{T} \cdot {\mathbf{E}},$$

where **T**, **S**, **E**, and **D**, are mechanical stress, mechanical strain, electrical field, and electrical displacement, respectively. Also, *s*^*E*^, *d*, and *ε*^*T*^ are elasticity matrix, coupling matrix, and dielectric permittivity matrix, respectively. Transposed matrix of *d* is defined as *d*^*t*^. Equation (4) describes the reverse piezoelectric effect where Eq. (5) indicates the direct piezoelectric effect^19^. Mass density of PVDF is equal to 1780 kg/m^3^ and the damping ratio has been considered equal to *η* = 0.01. The other parameters are as follows:

$$\begin{aligned} s^{E} & = \left[ {\begin{array}{*{20}c} {3.8} & {1.9} & {0.9} & 0 & 0 & 0 \\ {1.9} & {3.8} & {0.9} & 0 & 0 & 0 \\ {0.9} & {0.9} & {1.2} & 0 & 0 & 0 \\ 0 & 0 & 0 & {0.7} & 0 & 0 \\ 0 & 0 & 0 & 0 & {0.9} & 0 \\ 0 & 0 & 0 & 0 & 0 & {0.9} \\ \end{array} } \right]\,[{GPa}] \\ d & = \left[ {\begin{array}{*{20}c} 0 & 0 & 0 & 0 & 0 & 0 \\ 0 & 0 & 0 & 0 & 0 & 0 \\ {0.024} & {0.001} & { - 0.027} & 0 & 0 & 0 \\ \end{array} } \right]\,[{C/m}^{{2}} ] \\ \varepsilon_{r}^{T} & = \left[ {\begin{array}{*{20}c} {7.4} & 0 & 0 \\ 0 & {9.3} & 0 \\ 0 & 0 & {7.6} \\ \end{array} } \right] \\ \end{aligned}$$

where $\varepsilon_{r}^{T}$ is relative permittivity matrix.

**Figure 5:**
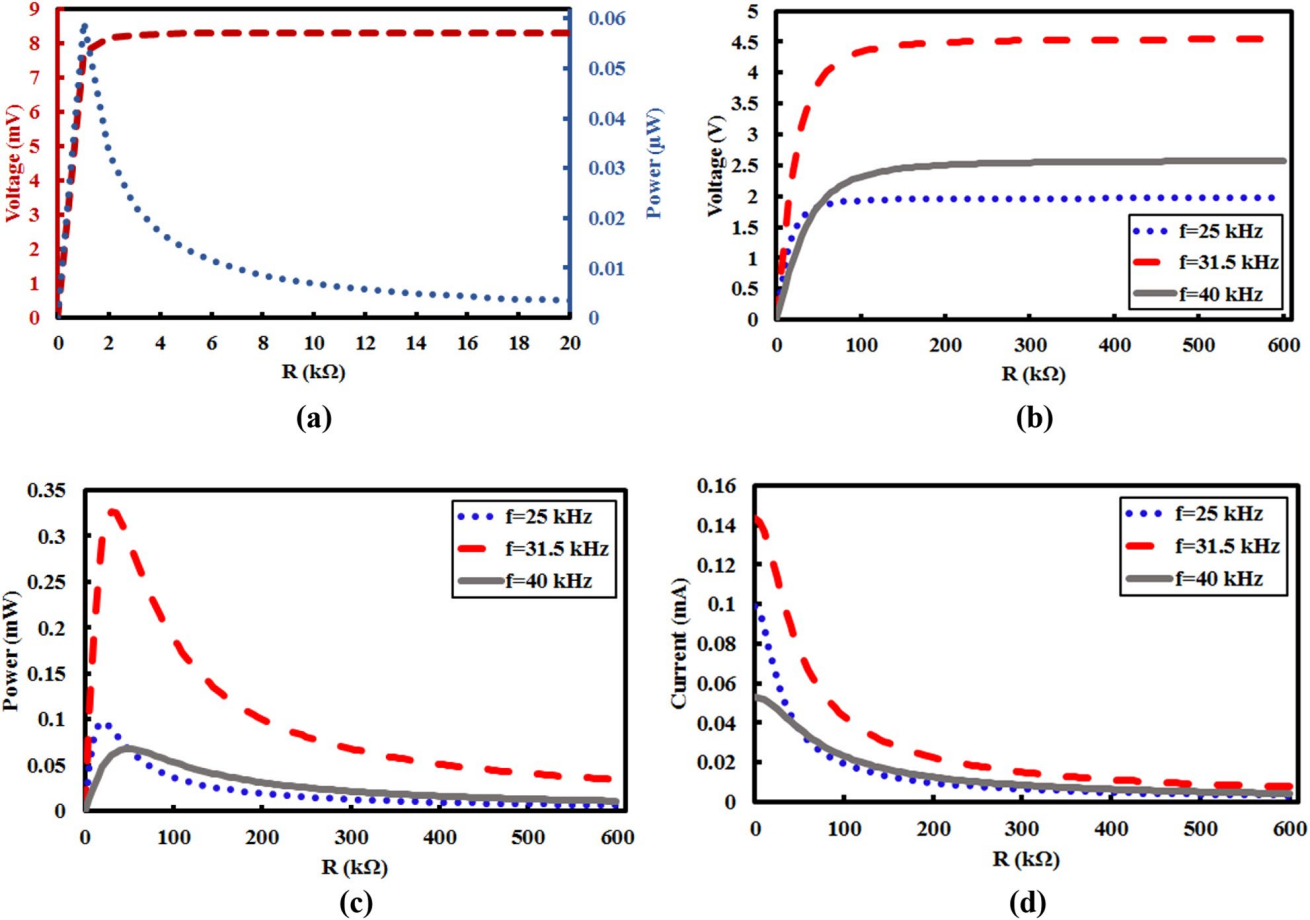
(**a**) Generated power and voltage in bare case, and generated (**b**) voltage, (**c**) power, and (**d**) current in working frequencies.

High confinement of elastic waves which are received from fiber core make more displacement on the piezoelectric energy harvester. The electrical voltage, current and power achieved from the proposed energy harvesting structure have been shown in Fig. 5.

According to Fig. 5a, it is clear that an insignificant power and voltage have been produced without any phononic crystal structure. However, in presence of phononic crystal fiber, output power has enhanced up to 800 times (in worst case) in comparison with bare case. Maximum value of power has been produced in *R* = 20 kΩ, *R* = 30 kΩ, and *R* = 50 kΩ for frequency of 25 kHz, 31.5 kHz, and 40 kHz, respectively. Hence, the phononic crystal fiber confines input waves with very high quality in the core and transmits them to the energy harvesting material. In this way, energy harvesting by phononic crystal fiber is realized.

## Discussions

**Figure 6:**
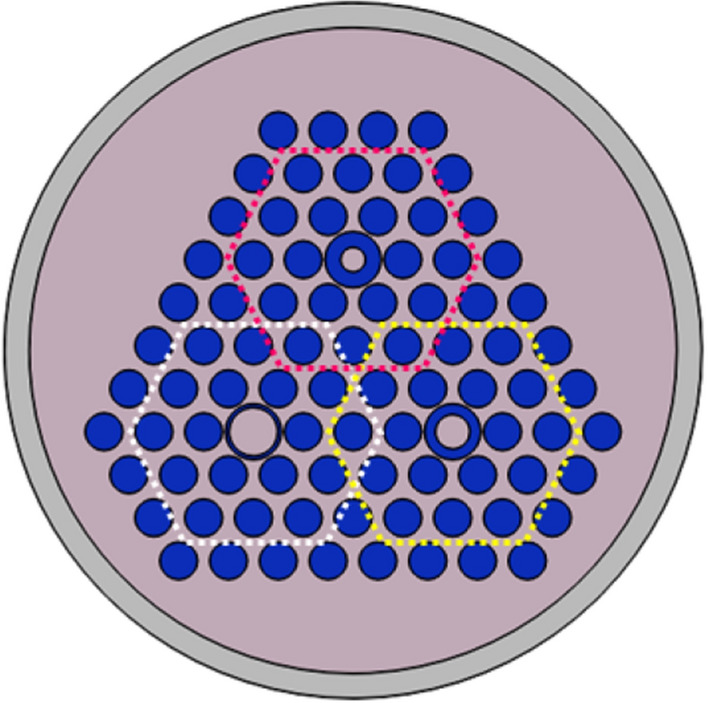
Cross-section of proposed multicore phononic crystal fiber with three different cores.

In order to integrate all separate phononic crystal fibers, we propose a multicore phononic crystal fiber which in it, three core have been formed of a tungsten hollow cylinder with internal radii of r_1_, r_2_, and r_3_. Proposed multicore phononic crystal fiber has been illustrated in Fig. 6.

**Figure 7:**
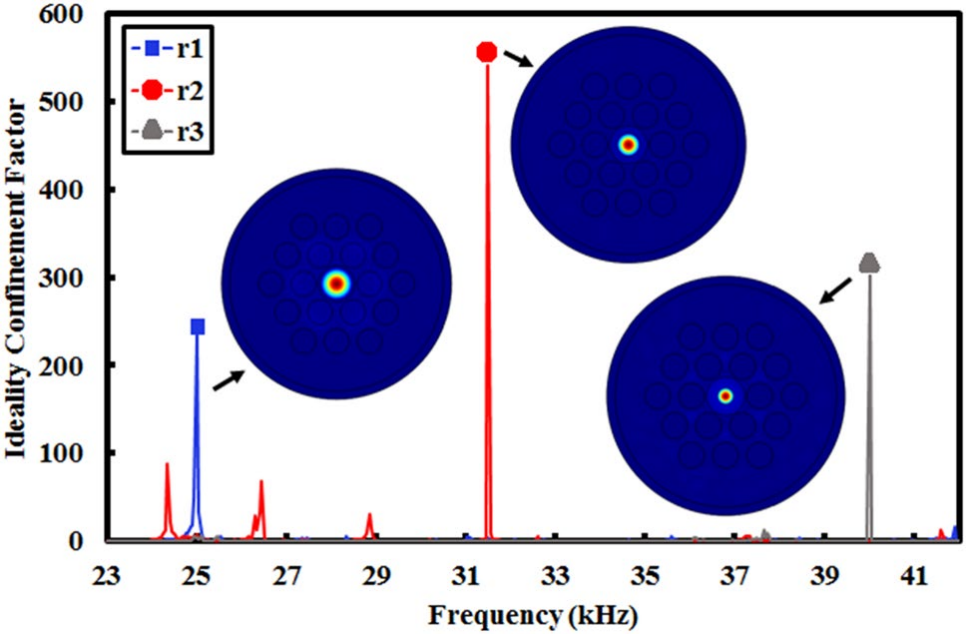
The curve of confinement factor versus frequency variations.

In order to obtain the multicore phononic crystal fiber, independent performance of each single-core phononic crystal fibers should be investigated. Hence, an ideality factor to indicate and calculate the confinement capability has been defined as Eq. (6), and named as ideality confinement factor.

6 $$Ideality\,confinement\,factor = \,\frac{{U_{core} }}{{U_{cladding} }}$$

where *U*_*core*_ indicates maximum displacement in the core and *U*_*cladding*_ illustrates the maximum displacement in the surroundings. In order to show independent performance of single-core phononic crystal fibers, variations of ideality confinement factor have been investigated versus different frequencies in three designed elastic fibers and achieved data has been depicted in Fig. 7.

**Figure 8:**
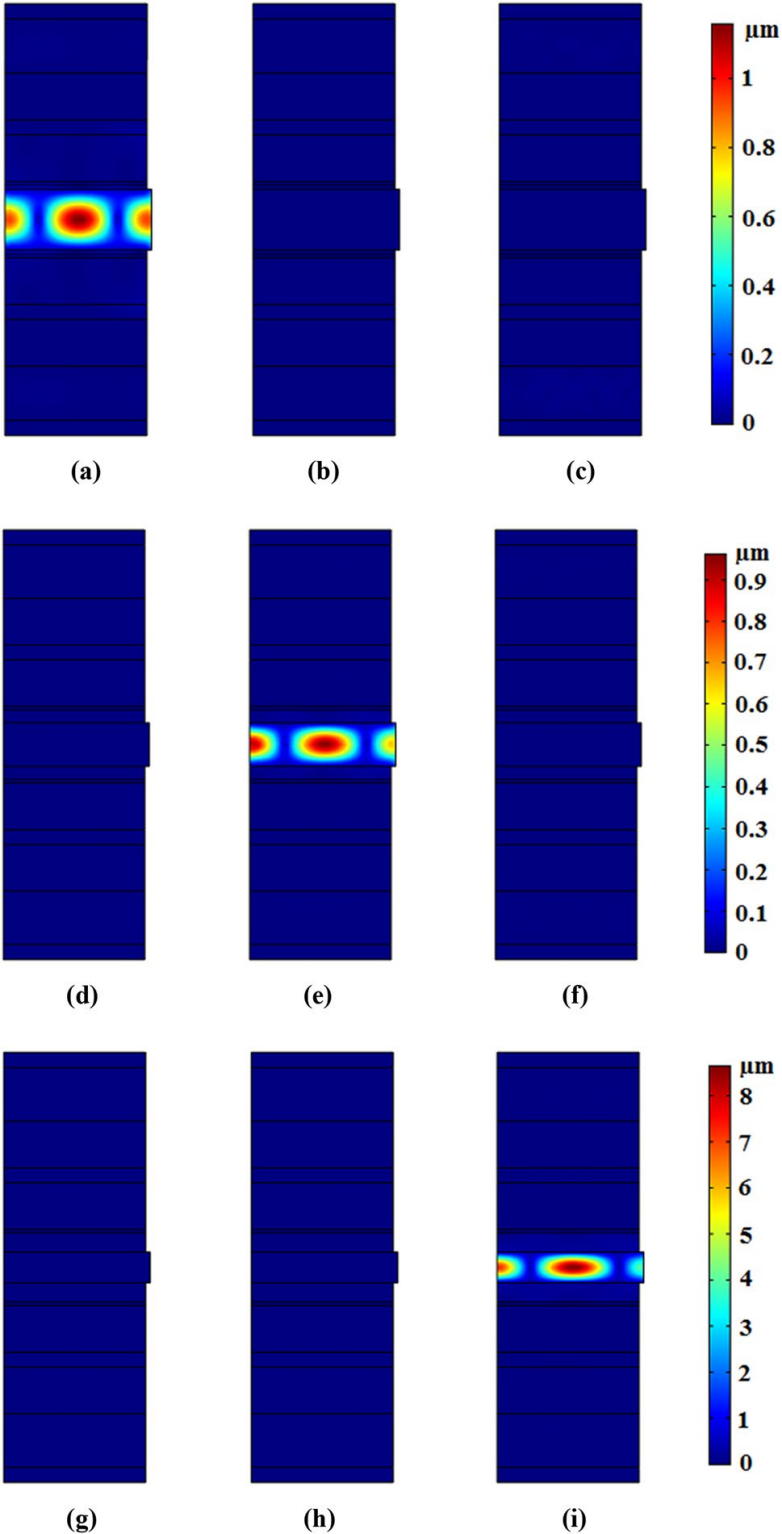
Displacement field on z–x plate in phononic crystal fibers with core radius equals to r_1_ and input frequency of (**a**) 25 kHz, (**b**) 31.5 kHz, and (**c**) 40 kHz, with core radius equals to r_2_ and input frequency of (**d**) 25 kHz, (**e**) 31.5 kHz, and (**f**) 40 kHz, with core radius equals to r_3_ and input frequency of (**g**) 25 kHz, (**h**) 31.5 kHz, and (**i**) 40 kHz.

Regarding to Fig. 7, it can be seen that maximum confinements are related to frequencies of 25 kHz, 31.5 kHz, and 40 kHz. In other words, applied acoustic waves with frequency of 25 kHz (for example) are confined in elastic fiber with core radius of r_1_. To more clarification of this concept, Fig. 8 has been shown.

Figure 8 is a graphical expression of extinction ratio which can be defined as $Ex.\,R.\,(dB) = \,10\log_{10} (U_{low} /U_{High} )$^8^ where *U*_*High*_ indicates the displacement of wave confined in desired port, and *U*_*low*_ is the displacement at the other undesired ports. The extinction ratio for designed elastic fibers is equal to − 23 dB in the worst case. Another important quality index of fibers is their longitudinal loss. By considering damping ratio for PMMA, *η* = 0.02^22^, the longitudinal loss is almost achieved equal to 0.9 dB/km.

Obtained results of single-core phononic crystal fibers show the fact that integration of single-core fibers is possible.

## Conclusions

In this investigation, we have designed phononic crystal fibers for energy harvesting application in metalworking factories. Each of single core fibers includes a tungsten hollow cylinder with same external radius and different internal radius. Incident waves with central frequencies from 25 to 40 kHz of 1/3 octave band are confined in proposed elastic fibers and transmitted to electrical energy harvesting station. By utilizing of a piezoelectric film at the end of cores, mechanical energy is converted to electrical energy. High capability of elastic fiber to confine the applied waves makes power generated up to 800 times versus bare case. The most important role of phononic crystal fiber in this application is transmitting the confined waves to desired location without significant loss. Maximum value of extinction ratio is equal to − 23 dB.

## Data Availability

The data that support the findings of this study are available from the corresponding author upon reasonable request.
